# The Visit of French Medical Men to London

**Published:** 1904-10-15

**Authors:** 


### THE VISIT OF FRENCH MEDICAL MEN TO LONDON.

Our visitors from France have shown since their arrival the energy and vivacity characteristic of their nation. On Tuesday, after an early visit to the French Hospital, a number proceeded to the Imperial Institute to inspect the physiological laboratories of the University of London. After being shown through the various departments of the laboratory, special attention being first paid to the work in electro-physiology, a demonstration was given with Dr. Waller's and Dr. Dubois' mechanical appliances for the percentage administration of chloroform. During the same morning members of the party visited the Royal College of Surgeons and several of the large hospitals, including the Charing Cross Hospital, King's College Hospital, and the Royal London Ophthalmic Hospital. Nor were the Poor-law institutions neglected, one section making their way, in spite of the dense fog which prevailed, to St. Mary (Islington) Infirmary. At noon the company were entertained at a déjeuner given by Mr. Butlin at the Whitehall rooms, after which a further inspection of various medical institutions was made. It is to be hoped that our friends from France will find as much benefit from their visit as the medical profession in this country will gain by their shrewd criticisms and energetic example.

